# Multiple shRNA combinations for near-complete coverage of all HIV-1 strains

**DOI:** 10.1186/1742-6405-8-1

**Published:** 2011-01-13

**Authors:** Glen J Mcintyre, Jennifer L Groneman, Yi-Hsin Yu, Anna Tran, Tanya L Applegate

**Affiliations:** 1 Johnson and Johnson Research Pty Ltd, Level 4 Biomedical Building, 1 Central Avenue, Australian Technology Park, Eveleigh, NSW, 1430, Australia

## Abstract

**Background:**

Combinatorial RNA interference (co-RNAi) approaches are needed to account for viral variability in treating HIV-1 with RNAi, as single short hairpin RNAs (shRNA) are rapidly rendered ineffective by resistant strains. Current work suggests that 4 simultaneously expressed shRNAs may prevent the emergence of resistant strains.

**Results:**

In this study we assembled combinations of highly-conserved shRNAs to target as many HIV-1 strains as possible. We analyzed intersecting conservations of 10 shRNAs to find combinations with 4+ matching the maximum number of strains using 1220+ HIV-1 sequences from the Los Alamos National Laboratory (LANL). We built 26 combinations of 2 to 7 shRNAs with up to 87% coverage for all known strains and 100% coverage of clade B subtypes, and characterized their intrinsic suppressive activities in transient expression assays. We found that all combinations had high combined suppressive activities, though there were also large changes in the individual activities of the component shRNAs in our multiple expression cassette configurations.

**Conclusion:**

By considering the intersecting conservations of shRNA combinations we have shown that it is possible to assemble combinations of 6 and 7 highly active, highly conserved shRNAs such that there is always at least 4 shRNAs within each combination covering all currently known variants of entire HIV-1 subtypes. By extension, it may be possible to combine several combinations for complete global coverage of HIV-1 variants.

## Introduction

HIV is characterized by high sequence variability with many hundreds of genetically unique strains [1,2]. These are classified based on changes in the viral envelope with 3 groups (M, N, and O) and several subtypes (or clades). There is a geographical clustering for each, with group M the main grouping globally and clade B the most common subtype in USA and Europe [2]. The only effective way to currently treat HIV is with the simultaneous use of multiple antiretroviral drugs to prevent the emergence of drug-resistant strains [3]. RNA interference (RNAi) is a recently discovered mechanism of gene suppression that has received considerable attention for its potential use in gene therapy strategies for HIV (for review see [4-6]). Expressed short hairpin RNA (shRNA) effectors are well suited for potential use in gene therapy. Sharing structural similarities to natural microRNA, shRNA consists of a short single stranded RNA transcript that folds into a 'hairpin' configuration by virtue of self-complementary regions separated by a short 'loop' sequence. shRNA is commonly expressed from U6 and H1 pol III promoters. These promoters are compact, active in many tissues, and are well suited to shRNA expression due to their relatively well-defined transcription start and end points. Importantly, pol III based shRNA expression cassettes have been incorporated into viral vectors which have been stably integrated both in culture and whole animals with effective silencing maintained over time [7-9].

**Figure 1:**
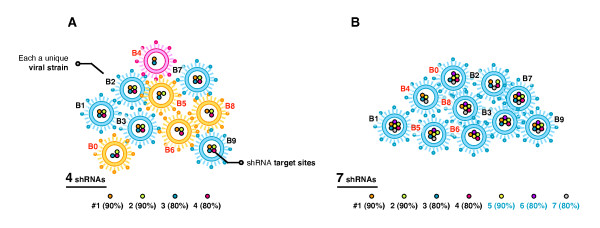
**More than 4 shRNAs are needed to obtain 4 matched to all variants**. Models predict that a minimum of 4 shRNAs is needed to prevent the emergence of viral escape mutants, however, this requires 4 shRNAs to be matched to all viral variants spanning the different subtypes. Unless all 4 shRNAs are 100% conserved across all targeted strains (unlikely) (**A**), then *more *than 4 shRNAs will be required to attain at *least *4 matched to each different strain (**B**). By studying the intersecting conservations of combinations as a whole, combinations can be assembled such at at least 4 shRNAs (4+) for a given combination (of > 4 shRNAs) are matched to all relevant strains.

The potency of individual shRNA has been extensively demonstrated in culture and there are now several hundred identified targets and verified shRNAs for HIV [10-12]. However, single shRNAs can be rapidly overcome by viral escape mutants possessing small sequence changes that alter the structure or sequence of the targeted region [13-16]. A combinatorial RNAi approach using multiple shRNAs is required to prevent the emergence of resistant strains [17-19], with models predicting that as few as 4 shRNAs will be sufficient [19,20]. However, this requires all 4 shRNAs to be matched to each of the 100's of circulating viral variants spanning the different subtypes. Previously reported combinations have shown much promise in laboratory tests, though a number are of limited clinical relevance in terms of target sequences [11,16,21-23]. This is because they tend to be assembled on the basis of the individual conservations of the component shRNAs without consideration of the intersecting conservation of the entire combination, where the highest individual conservations are not necessarily reflected in the intersecting conservation. This is an important point, as some strains will be (inadequately) covered by fewer than the intended number of shRNAs, thus facilitating the emergence of escape mutants. Unless all 4 shRNAs are effectively conserved across all targeted strains (unlikely), then *more *than 4 shRNAs will be required to attain at *least *4 matched to each different strain (Figure 1).

There are a number of potential methods for co-expressing multiple shRNA, including: multiple expression vectors [9,24,25], multiple expression cassettes from a single vector [11,26,27], and long single transcripts composed of an array of multiple shRNA domains [16,21,28-30]. The latter strategy is advantageous for gene therapy as it uses the fewest promoters, is the most compact and can be designed to mimic natural polycistronic miRNA clusters [22,30-32]. However, it is also the most difficult to currently use with many design variations and no clear guidelines. We and others have found that the original suppressive activities of the component shRNAs were not necessarily maintained in combination, and combinations of more than 2 shRNAs became increasingly difficult to assemble [16,28,29,33]. Moreover, effective combinations may be limited to only 3 domains, which is too few [34]. The multiple cassette strategy is a most useful method for immediate use due to its ease of design, assembly, and direct compatibility with pre-existing active shRNA. Others have also used this co-expression strategy to investigate multiple shRNA treatments for viral diseases, using cassette combinations ranging from 2 to 6 [11,26,27,35,36].

The primary aim of this study was to mathematically assemble and select combinations of highly-conserved anti-HIV shRNAs to target a maximum number of viral variants whilst minimizing the risk of selecting for escape mutants. We also aimed to characterize the intrinsic individual and combined suppressive activities of the component shRNAs when simultaneously expressed. We made 26 combinations of 2 to 7 shRNA with some containing at least 4 shRNA fully matched to 100% of clade B sequences, and up to 87% of all other clades. We found that while all combinations had high combined suppressive activities, the individual activities of the component shRNAs could vary compared to the corresponding single shRNAs. Importantly, we present a method by which highly relevant combinations can be selected, and have shown that a surprisingly small number of shRNAs can combined into single combinations with the potential for targeting entire subtype groups.

## Results

### A selection of anti-HIV shRNA

We have previously analyzed over 8000 unique 19 nucleotide (nt.) HIV-1 targets, and calculated their level of conservation amongst almost 38000 HIV gene sequence fragments containing 24.8 million 19 mers [12]. We characterized 96 in detail, and 10 of these, spanning 7 genes, were selected for assembly into combinations here (#0 - 9) (Table 1). This selection was based on a combination of activity, conservation and target diversity, with a bias towards selecting highly conserved sequences. The selected shRNAs were either active (> 50% activity) or highly active (> 75% activity), and the average conservation for the central cores (the first 19 nt. of each stem) was 74% amongst all known sequences and 85% for clade B subtypes. Our estimates of conservation were as stringent as possible in that we only regarded shRNAs that were fully matched as conserved (i.e. no mismatch tolerance). It may well be that our shRNAs are active against an even greater number of variants than we predict as some shRNAs can retain partial or full activity with some degree of mismatch to their targets. It is interesting to note that the two LTR shRNAs (#0 and 1) we chose were 100% conserved in clade B subtypes. It is also interesting that whilst our selection process was entirely independent of prior studies, several of our selected shRNA target sites (e.g. #3, 4 and 7) are highly similar to those identified by others; see our earlier report for a relevant list [11,12,37].

**Table 1:** The 10 shRNAs

#^a^	Target	-2 -1^b^	19 nt. core + 1/2 nt.^c^	Loop^d^	T.^e^	B^f^	ALL^g^
**0**	LTR 510-21	**AA**	CCCACTGCTTAAGCCTCAA**TA**	**A**CTCGAG**A**		**100**	70
**1**	LTR 527-21	**TC**	AATAAAGCTTGCCTTGAGT**GC**	**A**CTCGAG**A**	G	**100**	**93**
**2**	Gag 533-20	**AG**	GAGCCACCCCACAAGATTT**A**	**T**CTCGAG**T**		**80**	70
**3**	Pol 248-20	**AG**	GAGCAGATGATACAGTATT**A**	**C**CTCGAG**C**		**87**	**80**
**4**	Pol 2670-21	**GC**	AGTACAAATGGCAGTATTC**AT**	**A**CTCGAG**A**	G	74	73
**5**	Pol 2878-20	**AA**	GGTGAAGGGGCAGTAGTAA**T**	**T**CTCGAG**T**		**80**	74
**6**	Vif 9-21	**AA**	CAGATGGCAGGTGATGATT**GT**	**A**CTCGAG**A**		**92**	71
**7**	Tat (x1) 140-21	**CT**	ATGGCAGGAAGAAGCGGAG**AC**	**A**CTCGAG**A**	A	77	73
**8**	Vpu 143-20	**AA**	GAGCAGAAGACAGTGGCAA**T**	**C**CTCGAG**C**		**83**	66
**9**	Env 1428-21	**AA**	TTGGAGAAGTGAATTATAT**AA**	**A**CTCGAG**A**		**81**	73

^a ^shRNA reference number (**#**) used in this study.

^b ^The two nt. immediately upstream of the 19 bp core target site were taken into consideration when estimating *individual shRNA *conservations but *not *included in the shRNA stem, nor used in calculating intersecting conservations.

^c ^The shRNAs had 20 or 21 bp stems built around a 19 bp core placed at the base terminus of the shRNA which was extended by 1 or 2 nt. with target matched sequence at the loop terminus, as indicated in bold (and used when estimating *individual shRNA *conservations).

^d ^If the core was made into a 20 bp hairpin stem, then the last nucleotide of the loop was selected to be the complement of the last nucleotide of the p+2 position so that if the processed siRNA product(s) included the the last nucleotide of the loop then it too would be matched to the target (indicated by underline).

^e ^The shRNAs in which the last base of the anti-sense stem was 'T' also included a 'termination spacer' so as to prevent premature termination via an early run of 'T's. This nucleotide was always the complement of the first nucleotide of the p-1 position (but never a 'T'), so that if included in the processed siRNA product(s) it was also matched to the target.

^f ^% conservation for the 19 bp core in LANL clade B sequences only.

^g ^% conservation for the 19 bp core in ALL LANL sequences, irrespective of clade.

### Transferring shRNAs and confirming target-specificity

**Figure 2:**
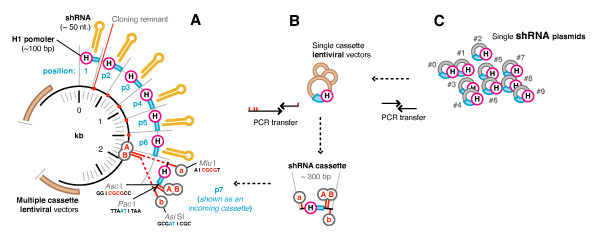
**An infinitely expandable cloning strategy**. In the example of our multiple cassette cloning strategy shown, a 7th cassette is being inserted into a vector that already has 6 cassettes integrated (**A**). The incoming donor fragment is a PCR amplified shRNA expression cassette (**B**) digested with 'a' (*Mlu I*) and 'b' (*Asi SI*) restriction enzymes (REs) which is ligated to the recipient vector opened up with 'A' (*Asc I*) and 'B' (*Pac I*) REs destroying the original 'a', 'A', b', and 'B' sites in the process. The newly created vector has the 'A' and 'B' sites reconstituted via the incoming donor fragment, ready for insertion of subsequent cassettes. Each shRNA expression cassette included the H1 promoter, shRNA, terminator and some flanking sequence to a total length of ~ 270 - 300 bp. (**C**) All 10 single shRNA expression cassettes were first transferred from pSilencer type plasmids (as assembled in prior work) as complete expression cassettes into single shRNA Lentivirus transfer plasmids (setup with an infinitely expandable MCS as detailed above).

**Figure 3:**
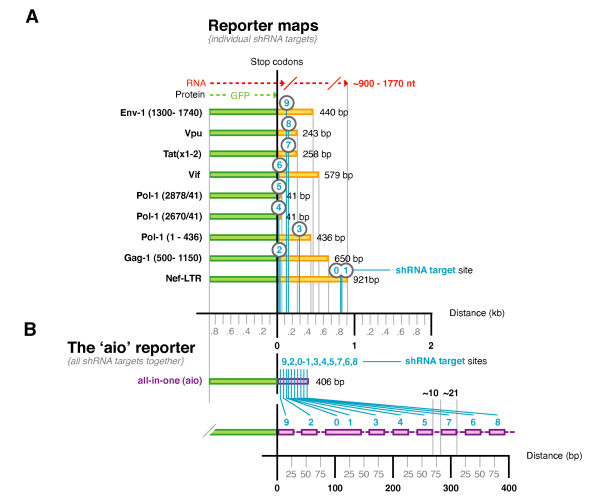
**Reporter maps**. (**A**) Each reporter contained GFP fused upstream to one of the accessory genes (for shRNAs #0, #1, #6, #7, and #8), a fragment of the core genes (#2, #3, and #9) or a small shRNA-specific target domain (#4 and #5) with stop codons placed between the two domains. Thus, each reporter produced a fused mRNA target composed of GFP plus the HIV-1 sequence from which only the GFP domain was translated. This was engineered to remove the possibility of HIV-1 protein products affecting shRNA activity. (**B**) We made an all-in-one reporter (the aio sense reporter) to measure the combined activity of simultaneously expressed shRNAs. It had a ~ 400 bp target domain composed of 8 sections of ~ 40 bp covering each ~ 20 bp shRNA target site plus ~ 10 bp either side, and one slightly longer shared section for the two LTR targets since they overlapped each other. Two more reporters were also made (though not shown schematically): the reverse complement of the aio sense reporter (the aio anti-sense) and a non-matched control reporter composed of 7 similarly sized target domains that were unmatched to the chosen shRNAs.

**Figure 4:**
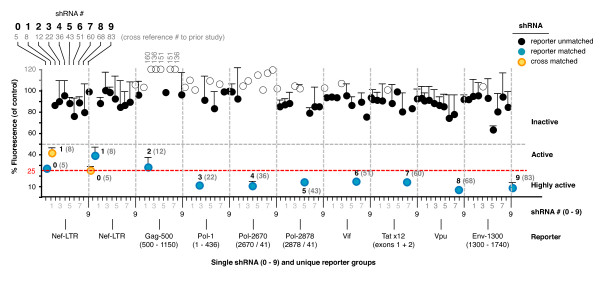
**shRNA specificity and activity was maintained in transferred expression cassettes**. Each shRNA expression plasmid was co-transfected into HEK293a cells with two reporters; the corresponding target-specific GFP fusion and a non-specific AsRed-1 fusion. All shRNA were separately tested with the 9 reporters individually matched to each shRNA (n.b. two LTR shRNA, #0 and #1, shared targeted the same reporter). Target-specific fluorescence levels were normalized to non-specific effects measured with the AsRed-1 fusion, and presented relative to the fluorescence levels from the corresponding empty expression cassette plasmid (value set at 100%; not shown). A key to the reference #s used in the original study is given below the 0 - 9 #s used here for cross-reference. Off-scale values (> 100%, i.e. no activity) are indicated by open circles without error bars, and with text labels where appropriate. Error bars are 95% Confidence Intervals (CI) from 3 independently repeated experiments.

Our 10 shRNAs were first transferred from pSilencer type plasmids [12] as complete expression cassettes (H1 promoter, shRNA region and terminator) into our Lentivirus transfer plasmid setup with an infinitely expandable MCS [38]. This setup enables any number of PCR/sub-cloned cassettes to be sequentially inserted by using restriction enzymes (REs) that are repeatedly destroyed and simultaneously re-introduced with each round of cloning (Figure 2). The transferred shRNA were each assayed for suppressive activity using 9 different fluorescent reporters matched to each shRNA (n.b. the two overlapping LTR shRNAs, #0 and #1, shared the same reporter) (Figure 3a). This was to confirm the specificity of each shRNA to enable us to accurately test the individual activities of simultaneously expressed shRNAs without reporter cross-reactivity. Each shRNA expression plasmid was co-transfected into HEK293a cells with two reporters; the corresponding target-specific GFP fusion and a non-specific AsRed-1 fusion. Target-specific fluorescence was measured 48 hours later, normalized to the fluorescence of the non-specific reporter, and activities were calculated relative to the fluorescence levels of a control plasmid with an empty expression cassette (composed of the H1 promoter, but no shRNA). All transferred shRNA maintained a comparable level of target-specific activity to that measured previously from the original plasmids, without reporter cross-reactivity (except that expected for #0 and #1) (Figure 4).

### Selecting combinations to maximize intersecting conservation

We mathematically assembled the 10 chosen shRNAs into all possible combinations of 4, 5, 6 and 7 different shRNAs with disregard to order. The total number of combinations (k) from a given set size (n) can be found by the combinatorial 'choose function': n!/(k!(n - k)!). For example, 10!/(4!(10-4)!) equalled 210 possible combinations of 4 shRNAs from our selected set of 10. There were 252 possible combinations of 5 shRNAs, 210 of 6, and 120 of 7. For each combination we calculated the intersecting conservations, from *at least 4 *of the component shRNAs, using the first 19 bp of each shRNA stem in accord with our previous target conservation profiling method [12]. Intersecting conservations were calculated using 1224 HIV-1 genome sequences, some with incomplete LTR sequence, obtained from the Los Alamos National Laboratory in 2007 (LANL; http://www.hiv.lanl.gov). We created 4 sub sets from these: all clade B sequences (229 sequences), all other clades (995), clade B sequences that contained sufficient LTR sequence to analyze combinations including our LTR shRNAs (127), and all other clades with sufficient LTR sequence (549).

There was generally poor intersecting conservations from combinations of 4 shRNAs. Several combinations of 5 had at least 4 shRNAs (4+) conserved in 89 - 97% of clade B sequences, though only 40 - 58% in all other subtypes (Table 2). But some combinations of 6 and 7 shRNAs had 4+ intersecting conservations of 98 - 100% in clade B and 65 - 87% in all other subtypes. We selected 17 combinations to construct, composed of 6 combinations of 5× shRNAs, 8 combinations of 6× shRNAs, and 3 combinations of 7× shRNAs. Our selection included combinations that excluded LTR shRNAs as there is still some uncertainty surrounding the accessibility of the incoming virus and the LTR as an *in-vivo *RNAi target [39-41]. Though given the possibility [37,39,40], we also selected combinations that specifically included LTR shRNAs as they had the highest individual conservation levels. Combinations including overlapping shRNA targets (e.g. shRNAs #0 and #1) were discounted. The shRNA order of each combination was chosen to minimize construction steps by creating common sub-combinations from the most common shRNAs first. In total, there were 26 combinations assembled including 9 sub-combinations of 4 or less shRNAs required for our 17 final combinations of 5 to 7 shRNAs.

**Table 2:** Percentage conservations for the combinations

		229 sequences				995 sequences				127 sequences				549 sequences			
		
		Clade B^d^				Others^e^				Clade B (LTR)^f^				Others (LTR)^g^			
#^a^	**Combin**.^c^	4+	5+	6+	7+	4+	5+	6+	7+	4+	5+	6+	7+	4+	5+	6+	7+
**5.1**	3.4.7.2.9	**89**	48	-	-	54	15	-	-	**91**	53	-	-	58	15	-	-
**5.2^b^**	3.4.7.2.0	n/a	n/a	-	-	n/a	n/a	-	-	**94**	57	-	-	57	23	-	-
**5.3**	3.8.9.2.7	**92**	49	-	-	40	13	-	-	**94**	54	-	-	41	15	-	-
**5.4^b^**	3.8.5.9.0	n/a	n/a	-	-	n/a	n/a	-	-	**95**	64	-	-	58	20	-	-
**5.5**	3.8.5.2.9	**92**	53	-	-	48	12	-	-	**94**	59	-	-	54	13	-	-
**5.6^b^**	3.8.5.2.1	n/a	n/a	-	-	n/a	n/a	-	-	**97**	65	-	-	53	10	-	-
**6.2^b^**	3.4.7.2.0.5	n/a	n/a	n/a	-	n/a	n/a	n/a	-	**100**	**89**	51	-	**81**	50	15	-
**6.3**	3.8.9.2.7.6	**100**	**86**	46	-	65	26	4	-	**99**	**89**	49	-	67	23	3	-
**6.4^b^**	3.8.5.9.0.6	n/a	n/a	n/a	-	n/a	n/a	n/a	-	**100**	**88**	60	-	75	38	5	-
**6.5^b^**	3.8.5.2.9.0	n/a	n/a	n/a	-	n/a	n/a	n/a	-	**99**	**94**	58	-	**80**	45	13	-
**6.6**	3.8.5.2.9.7	**99**	**87**	43	-	68	31	9	-	**98**	**92**	47	-	73	34	10	-
**6.7^b^**	3.8.5.2.1.7	n/a	n/a	n/a	-	n/a	n/a	n/a	-	**100**	**94**	53	-	75	30	7	-
**6.8^b^**	3.4.7.2.0.6	n/a	n/a	n/a	-	n/a	n/a	n/a	-	**99**	**88**	52	-	77	40	5	-
**6.9**	3.4.7.2.9.5	**98**	**84**	43	-	75	45	9	-	**98**	**87**	47	-	79	50	10	-
**7.3^b^**	3.8.9.2.7.6.0	n/a	n/a	n/a	n/a	n/a	n/a	n/a	n/a	**100**	**98**	**89**	48	**87**	57	22	3
**7.5**	3.8.5.2.9.7.6	**100**	**99**	**81**	41	**84**	56	18	3	**100**	**98**	**84**	44	**85**	58	17	2
**7.7**	3.4.7.2.9.5.6	**100**	**96**	**80**	40	**86**	64	31	3	**100**	**94**	**83**	43	**86**	64	34	2

^a ^Combination reference number (**#**) used in this study.

^b ^As these combinations contained an LTR shRNA (0 or 1) the intersecting conservations calculated on all sequences (where a large number lacked LTR sequence coverage), were not applicable (**n/a**); only the LTR containing subset were used, as shown.

^c ^The component shRNAs (in order of arrangement) for each combination.

^d ^The clade B sequences only.

^e ^The sequences of all other clades (excluding clade B).

^f ^The subset of clade B sequences that contain LTR sequence coverage.

^g ^The subset of all other sequences (non B) that contain LTR sequence coverage.

### Establishing positional differences in combinations of up to 7 cassettes

**Figure 5:**
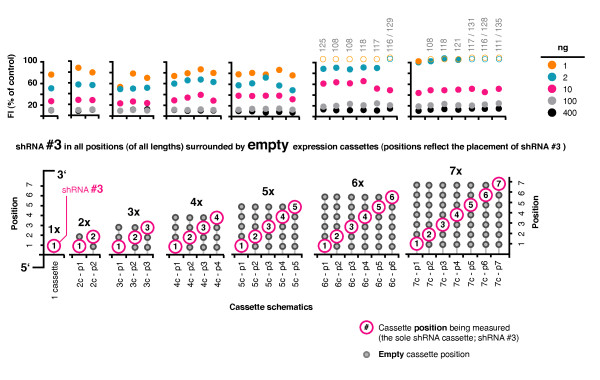
**Cassette number and position effects on suppressive activity**. Twenty seven control plasmids, each containing a single shRNA expression cassette (shRNA #3) plus 1 or more empty expression cassettes for all possible 2, 3, 4, 5, 6 and 7 cassette plasmids were tested with the fluorescent reporter assay using the matched Pol-1 (1 - 436) reporter across a titrated range of shRNA plasmid amounts from 400 ng to 1 ng. The activity of each combination was calculated as a% of the fluorescence from the corresponding control of same cassette number and ~ length but composed entirely of empty expression cassettes (values set at 100%; not shown). Off-scale values are indicated by open circles.

We also created 33 controls, composed of 6 empty cassette combinations of 2 - 7, and 27 combinations of a single shRNA (shRNA #3; Pol 248-20) surrounded by 1 or more empty expression cassettes (in combinations of 2 - 7). In this way our control shRNA could be tested in each position for potential effects by neighboring promoters without competition from other shRNAs for the RNAi machinery. The suppressive activity of the 27 plasmids was tested using the fluorescent reporter assay with the corresponding reporter (Pol-1), across a titrated range of shRNA plasmid amounts from 400 ng to 1 ng (Figure 5). There was a trend towards decreased activity from plasmids with increased cassette number, irrespective of cassette position, which was most apparent for the 6 and 7 cassette plasmids at low doses. Suitable activity was, however, maintained in all variants for the high to mid doses tested (400 - 100 ng), with respective standard deviations in apparent suppressive activity of 2% and 5% across all positions in all combinations. This confirmed that cassette order had no obvious effect on intrinsic suppressive activity.

### Individual activities measured under simultaneous expression

**Figure 6:**
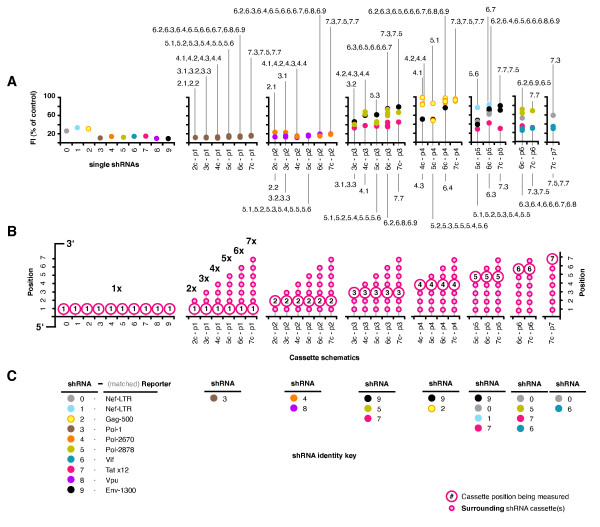
**Individual activities measured under simultaneous expression**. (**A**) Every combination and the 10 single shRNA plasmids were separately transfected with each matched fluorescent reporter corresponding to its component shRNAs to measure the individual suppressive activities of each shRNA when expressed simultaneously with others. Activities are plotted according to total cassette number and each cassette position (1 to 7) (**B**). Different combinations of identical cassette number are grouped in columns (e.g. the 6 combinations of 5 cassettes), shRNA # is indicated by color coding, and the combination number for individual points is indicated where points differed from the main cluster. The combination numbers present in each column are given, and also color coded to match the shRNA in the position being tested and the matched reporter used (**C**). For example, combinations 6.2 (3.**4**.7.2.0.5), 6.8 (3.**4**.7.2.0.6) and 6.9 (3.**4**.7.2.9.5) in **position 2 **were assayed with the Pol-2670 reporter (matched to shRNA **#4**), and combinations 6.3 (3.**8**.9.2.7.6) - 6.7 (3.**8**.5.2.1.7) were assayed with the Vpu reporter (matched to shRNA **#8**). Values shown are representative of 3 independently repeated experiments.

We measured the individual suppressive activity of each shRNA within all combinations when expressed simultaneously using our fluorescent reporter assay. Every combination and the 10 single shRNA plasmids were separately transfected with each matched reporter (Figure 6). Thus, the apparent suppressive activities likely reflect the individual suppressive contribution of each shRNA to the total. The activities from the single plasmids matched those seen previously. Likewise, the activity of shRNA #3 (the first position in all combinations) was similar for all combinations and the single shRNA plasmid. Activities from the second position shRNAs were also comparable to the single shRNA plasmids, however, all shRNA activities from position 3 onwards were notably reduced relative to the single shRNA plasmids. This was most obvious for shRNA #9 in positions 3, 4 and 5, and #1 in position 5; irrespective of total cassette number. Activities from each shRNA generally clustered, regardless of the position or length of the combination it was present in. For example, while the activities of combinations of 3 to 7 shRNAs measured for shRNA #7 in positions 3, 5 and 6 (i.e. measured with reporter Tat ×12) differed on average more than 2 fold from the #7 single shRNA, they had a standard deviation of only 4.8%. Our data suggests that shRNA competition may reduce the individual suppressive activities of simultaneously expressed shRNAs, with some sequences more susceptible than others.

### Building all-in-one reporters

We made a new **a**ll-**i**n-**o**ne reporter (the **aio **sense reporter) to measure the combined or total activity of all shRNAs within each combination acting in concert against a single target transcript. This reporter had a ~ 400 bp target domain composed of fused target sections for our 10 chosen shRNAs (Figure 3b). There were 8 sections of ~ 40 bp covering each ~ 20 bp target site plus ~ 10 bp either side, and one slightly longer shared section for the two overlapping LTR targets. Two additional reporters were also made. One was the reverse complement of the aio sense reporter (the aio anti-sense) designed to measure suppressive activity of the siRNA passenger strand derived from the anti-sense shRNA stem. The other was a non-matched control reporter composed of 7 similarly sized target domains that were unmatched to the chosen shRNAs. To go with this last reporter, we assembled a series of 7 corresponding non-matched single shRNA controls and used them to make 2, 3, 4, 5, 6 and 7 cassette control combinations. The sequences of the single shRNA controls were derived from the backwards sequence of shRNAs #3, #8, #9, #2, #7, #6 and #0. In this way they were unmatched to the aio reporters yet had identical nucleotide compositions (but in reverse order) to retain similar thermodynamic profiles.

### Combined activities measured with an all-in-one reporter

**Figure 7:**
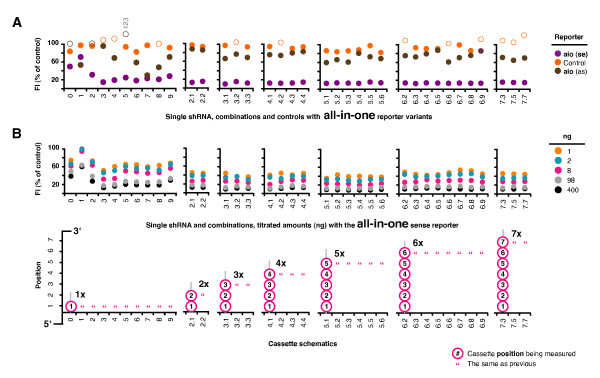
**Combined activities measured with an all-in-one reporter**. (**A**) All 10 single shRNA plasmids, all 26 combination plasmids, and the extra non-matched shRNA plasmids (singles plus **c**ontrol **c**ombinations (**c.c**.) - see Additional file 1 for control data) were separately transfected with the three all-in-one reporters; the aio sense (to measure specific activity of the intended guide strand), the aio anti-sense (to measure unintended activities from the expected passenger strand), and the non-matched control to measure potential non-specific effects from our combinations. Off-scale values (> 100%, i.e. no activity) are indicated by open circles and text labels where appropriate. (**B**) All single shRNA and combination plasmids were re-tested with the aio sense reporter using a titrated amount of shRNA or combination plasmid from 400 ng to 1 ng. In this example, the filler plasmid used to maintain a constant amount of DNA per transfection was our base lentivirus (backbone) plasmid, without any cassettes (i.e. competing H1 promoters). Values shown are representative of 2 or more independently repeated experiments.

All single shRNA, combinations, and non-matched control plasmids were separately transfected with all three reporters (Figure 7a) (Additional file 1 for the control data). The aio sense reporter activities for the 10 single shRNA differed slightly in magnitude to that seen with the previous reporters, but still followed the same relative pattern. shRNAs #0, #1 and #2 were the least active. Interestingly, the aio anti-sense reporter showed that shRNAs #1, #7 (especially) and #8 were being at least partly processed so that the passenger strand was being loaded into RISC. The activity of shRNA #1 was particularly poor, with the passenger strand exhibiting greater suppressive activity than the guide strand. The activities for all combinations of 2 through to 7 cassettes were similar to each other and the activities of the most active single shRNA (measured with the aio sense reporter). It may be that this was close to the highest silencing level achievable with the aio reporter under the current conditions of our assay system. There was no notable passenger strand activity from any combination. In all cases the single shRNAs and combinations exhibited no notable non-specific effects on the control reporter. The backwards control shRNAs showed the expected reverse trend, with no effect on either aio reporter, but some suppression of the corresponding control reporter. Activity levels were spread across the inactive to active classification groups as the backwards control shRNAs were neither designed nor previously selected for activity. Interestingly, the combined suppressive activities of these mediocre shRNAs at maximum dosage was not additive, i.e. resulting in greater total suppressive activity. Again, no control combination exceeded the activity of the most active single shRNA.

### Titrations to look at sub-saturating differences between combinations

We repeated the transfections of the aio sense reporter with each combination, but titrated the amount of single shRNA or combination plasmid from 400 ng to 1 ng to determine if there was a sub-saturating point at which larger combinations were more active than smaller ones (Figure 7b). The suppressive activities at the higher dosages were similar to that seen previously, and all combinations were more active than any of the single shRNAs at all titration points. This showed that one or more shRNAs in combinations can exhibit an increased combined suppressive effect at sub-saturating expression levels compared to any one of the component single shRNAs. However, there were no obvious differences between combinations of different number, with an average standard deviation of only 2% at all titration points.

**Figure 8:**
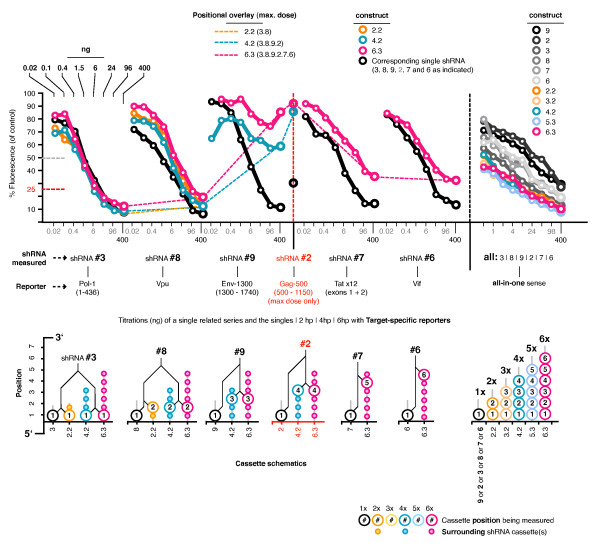
**Titrations of a select series of related combinations with individual reporters**. We measured the titrated activities for a single series of related combinations (2.2, 4.2 and 6.3) and their corresponding single shRNA plasmids (#3, #8, #9, #2, #7, and #6) using shRNA-specific reporters. We overlaid connection lines between the maximum dose values for each shRNA/reporter to show the positional relationship of each shRNA in the combinations for comparison to the equivalent measurements using the aio reporters. We expanded the titration points for the same series of plasmids tested with the aio sense reporter, and included them on the same scale for comparison. n.b. we were unable to test titrated amounts of shRNA #2 with the Gag-500 reporter due to stock contamination. Values shown incorporate representative data from 2 independent experiments.

We also measured the titrated activities using shRNA-specific reporters and a series of related combinations (2.2, 4.2 and 6.3) and their corresponding single shRNA plasmids (#3, #8, #9, #2, #7, and #6) (Figure 8). The activities of the selected single shRNAs at maximum dosage was generally higher with the shRNA-specific reporters (cf. the aio reporter), excepting #2, and clustered closely for both reporter types. The individual suppressive activities of each shRNA expressed simultaneously with all others was reduced relative to activities from the corresponding single shRNA plasmids at all titration points, with the exception of shRNA #3 (position 1). shRNAs #9 and #2 in the central positions (3 and 4) displayed notably impaired contributing activities when expressed in combination. The positional overlay connecting the maximum dosages showed the same pattern as seen previously when testing all combinations. Likewise, the combined activities for all combinations were similar at all titration points, but generally greater than the component single shRNAs at lower dosages.

## Discussion

In this study we aimed to mathematically assemble, select and test combinations of highly-conserved anti-HIV shRNAs to find those with the highest intersecting conservations of 4+ shRNAs across all known viral strains. Importantly, we have shown that it is possible, with careful consideration of the individual and intersecting conservations of combinations of shRNAs, to assemble shRNA combinations against entire subtypes. Even when selecting the most conserved individual shRNAs we were unable to identify a combination of 4 that was fully matched to all variants analyzed, as there were not 4 non-overlapping shRNAs that were 100% conserved. But with selected combinations of 7 shRNAs we could attain at least 4 shRNAs matched to 100% of clade B subtypes, and up to 87% of all other clades - a highly significant finding. In demonstration of the need to consider intersecting conservations, 5 of our highest individually conserved shRNAs (#0|1, 3, 6, 8, and 9) had an intersecting conservation for clade B subtypes that was 6% lower than other possible combinations (91 vs. 97%). Also, we found that different combinations were better suited to different subtypes. For example, the combinations of 5 shRNAs with the highest intersecting conservations for clade B subtypes (96 - 97%) had conservations for all other subtypes that were between at least 16 and 28% less (34 - 53%) than others most highly conserved for all other subtypes (62 - 69%). Thus, subtype-specific intersecting conservations should also be considered if tailoring combinations to localities with a subtype bias.

The data from our series of combinations containing a single copy shRNA #3 surrounded by empty expression cassettes revealed that although there was some reduction in activity for combinations of 6 and 7 shRNAs, there was no obvious difference in the activities of the different cassette positions for any combination length. When we assembled combinations of multiple shRNAs and tested the individual activities of the component shRNAs, we found that the shRNAs in positions 1 and 2 were the most active (#3, 4 and 8), and similar in activity to the corresponding single shRNAs for all combinations. However, shRNAs in positions 3 to 7 exhibited a marked reduction in activity compared to their single shRNA counterparts, irrespective of combination length or composition, and notably to similar levels for each shRNA. Some of the corresponding single shRNAs were highly active (#5, 6, 7 and 9), and just as active as the single shRNAs corresponding to positions 1 and 2. We conclude that the shRNAs located in positions 3 to 7 were likely more susceptible to a reduction in suppressive activity due to competition than the shRNAs in positions 1 and 2. If so, the effect of competition on individual suppressive activities may, at least in part, be sequence dependent. Future screens for component shRNAs for assembly in combinations may benefit from the inclusion of an additional test to measure individual suppressive activity when expressed in combination. However, it is also possible that there may be some additional positional influence contributing to our observations from the shRNA combinations that was not apparent from our shRNA #3 control series. This possibility could be examined with further work including the assembly of permutations of the combinations to place each shRNA in several different positions whilst being surrounded by other shRNAs.

Potential competition for the available RNAi machinery between simultaneously expressed shRNAs (and host cell miRNAs) also needs to be considered. It is likely that expression level will need to be carefully controlled to maximize the specific activities of the anti-HIV shRNAs, yet minimize the impact on the natural miRNA processes. Another potential issue is that the shRNAs not matched to the given strain may reduce the individual impacts of those that are matched [16,29], which implies that combination size should be kept to a minimum. We also noted from our positional testing experiments that combinations of > 5 shRNAs began to show reduced activity from all cassette positions due to increased size alone. It may be a more effective gene therapy strategy to use several smaller combinations of ~ 5 shRNAs selected with intersecting conservations specific to the strains that predominate geographical locations, rather than a single larger combination to cover all variants simultaneously.

Although multiple repeated expression cassettes is currently a most useful co-expression strategy for multiple shRNAs, it has some shortcomings, including concerns regarding long-term stability and efficacy [28]. We chose this system here not because we necessarily think that it will be the best in the long run, but because at the time of design it was the most advanced method compatible with out goals. We and others have found that large increases in vector size may affect vector production [23,33,42]. It is possible that the impact of these issues could be reduced or eliminated with fewer cassettes, further supporting the use of the smallest effective combination size. Current designs like ours that use unregulated pol III promoters may also have to be redesigned with regulated or inducible expression units for *in vivo *applications (e.g. [22]). Our choice of the H1 promoter (compared to say, the U6 promoter) was because of our extensive experience with this promoter and its common use for expressing shRNAs. Furthermore, there are reports that U6 expressed shRNAs may cause an adverse response *in vivo*, though this was overcome by using different pol II promoter types [43]. Other co-expression methods may be ultimately better suited for our gene therapy, such as single transcripts composed of multiple target domains. Several such designs have been tested by others, though in some cases it has been difficult to attain activity from more than 3 domains [34,44]. Also, their use will also likely be complicated by the probability that each domain is processed in a different manner to the component shRNAs. As with all co-expression methods, one should remain mindful of the possibility of unexpected off-target effects. Additionally, all uses of a lentivirus delivery system for a HIV sequence-specific treatment such as shRNA should, at some stage, consider the possibility that the shRNA used may interfere with the production of the lentivirus itself. We, however have not yet found this problematic [45] and the extent to which this would be a practical problem on a commercial-scale is as yet undetermined, though there are several ways in which it may be overcome.

We have since begun testing our combinations in HIV replication assays and discovered that repeat-mediated deletion can result in the loss of one or more complete expression cassettes [45]. We have extensively characterised the frequency of this phenomenon, and modeled its likely impact on treatment success. Surprisingly, we have found that even significant amounts of deletion are unlikely to affect the treatment outcome. Thus, the next step is to test the combinations for their capacity to suppress the emergence of escape mutants over time, similar to other studies [11,16]. This is the critical assessment of activity to confirm the theoretical advantages of multiple shRNAs frequently speculated upon [46-48], and supported by mathematical modeling [49]. In addition to this, the long-term stability of our combinations, and potential cellular affects will need to be assessed. Direct measurements of expression level could also be beneficial for refinement of our configurations and better understanding potential differences resulting from cassette positions (and how these operate over time under stable expression). Finally, reformulated combinations could be investigated using a wider range of shRNAs reanalyzed to include the following: (1) very highly active, e.g. > 95%, (2) tested suppressive activity when expressed in combination, and (3) high strand bias to reduce competition with the active strand. All these factors will be important to maximize the impact from combinations of the smallest effective size.

In summary, we have assembled the most clinically relevant shRNA combinations to date, and characterized the intrinsic suppressive activities of both the component shRNAs and the combinations as a whole. While we found all combinations had high combined suppressive activities, there were also large changes in the individual activities of the component shRNAs. Though a useful start, it is not the physical configuration of our multiple cassette method that is the key finding. Rather, we have demonstrated that it is possible to assemble combinations of 4+ highly active, highly conserved shRNAs that can cover all known variants for entire subtypes (e.g. clade B). Such combinations could thus be tailored to geographical sub-type prevalence with the theoretical potential to prevent the emergence of therapy-resistant strains. By extension, our work suggests that it may also be possible to assemble just a few combinations for complete global coverage of all known HIV-1 variants; something as yet unachievable by any other means.

## Methods

### Single target and all-in-one (aio) fluorescent reporter constructions

The HIV-1 fluorescent protein-target fusion reporter plasmids were constructed using EGFP (from pd4-d4EGFP-N1, BD Biosciences), AsRed1 (from pAsRed1-C1, BD Biosciences) and HIV-1 sequences PCR amplified or synthetically generated from variant NL4-3 [Genbank:AF324493] (Additional file 1). Each single target reporter contained GFP fused upstream to one of the accessory genes (for shRNAs #0, #1, #6, #7, and #8), a fragment of the core genes (#2, #3, and #9) or a small shRNA-specific target domain (#4 and #5) with stop codons placed between the two domains so that a fused mRNA target was transcribed but only the GFP domain was translated. The aio reporters were created using custom generated target domains (GenScript; http://www.genscript.com) composed of 9 target domains matched to our 10 shRNAs, or 7 unmatched control domains (for the control reporter) which were transferred via PCR into our EGFP reporter base plasmids. The elements of the 406 bp combined target domain synthesized for the aio sense reporter were (*Xho *I - *Bgl *II {*for cloning*} - #9 - #2 - #0|1 - #3 - #4 - #5 - #7 - #6 - #8 - *Sal *I - *Bam *HI {*for cloning*}).

### Assembly of multiple shRNA expression plasmids

The infinitely expandable multiple cloning site was assembled by annealing two complementary synthetic oligonucleotides, and inserting it into a recipient plasmid (a 7 kb carrier plasmid encoding a lentiviral transfer vector) which was a derivative of pKC(ro^-^)MND.MCS obtained from Cell Genesys, as previously described [38]. The 10 chosen shRNA targets, and the original individual shRNA expression plasmids used here as PCR templates were selected and constructed as previously described [12,50] (Additional file 1). The multiple shRNA combinations were assembled in our lentivirus plasmids by sequentially inserting each PCR amplified shRNA expression cassette. Because many shRNAs were present in several combinations, we were able to minimize construction effort by creating shared sub-combinations from the most common shRNAs first. As such, all combinations began with shRNA #3, and had either #4 or #8 in position 2, #5, #7 or #9 in position 3, and so on, becoming increasingly diverse for each combination. The PCR primers used to amplify the shRNA expression cassettes were common to all plasmids; the forward primer (5'-3'): GC {*seat*} ACGCGT {*Mlu I*} GTT TTC CCA GTC ACG AC {*binding site*}, and the reverse primer (5'-3'): GC {*seat*} GCGATCGC {*Asi SI*} TTAATTAA {*Pac I*} CCCGGG {*spacer*} GGCGCGCC {*Asc I*} GCT GCA ATA AA CAA GTT A {*binding site*}. Each PCR consisted of the core 2 primers (20 pmol each), 1× PCR II buffer (Roche) 2.5 mM MgCl_2_, 10 mM dNTPs (each), ~ 100 ng of template, 0.5 μl AmpliTaq-Gold (Roche), and H_2_O to a final volume of 50 μl. Each PCR was cycled at 1×: 94°C for 10 min., 35×: 94°C for 30 sec. | 55°C for 30 sec. | 72°C for 30 sec., and 1 × 72°C for 10 min. End digestions were conducted directly in the PCR mix (after cycling) by adding 5 μl of 10× BSA, 1 μl each of *Mlu *I and *Asi *SI and incubating @ 37°C for a minimum of 1 hr. All restriction enzymes were sourced from New England Biolabs (NEB). Digested cassettes were separated on 2% TAE agarose gels, gel extracted (Qiagen Gel Extraction kit) and eluted in 35 μl of H_2_O. Recipient plasmids were prepared by digestion of ~ 10 μg with 1 μl each of *Asc *I and *Pac *I, NEB 4 buffer, BSA plus H_2_O to a final volume of 50 μl and incubation at 37°C overnight. This was followed by heat inactivation (65°C for 20 min.) and de-phosphorylation by adding 5 μl Antarctic Phosphatase (NEB), 5 μl buffer and incubating at 37°C for a minimum of 1 hr. Antarctic Phosphatase was heat inactivated (65°C for 10 min) prior to separation on 1% TAE agarose gels and gel extraction (performed as already described). Single donor cassettes were ligated into the linearized recipient plasmid using 4 μl of vector, 6 μl of shRNA cassette, 10 μl of Quick DNA ligase buffer and 1 μl of Quick DNA ligase (NEB). The ligations were incubated at room temperature for 5 min., and then purified using the QIAgen PCR Purification kit by mixing with 5 volumes (105 μl) of Buffer PB, and eluting in 35 μl H_2_O. Ligated products were transformed by electroporation under standard conditions, and positive colonies were identified by a direct colony PCR technique using Pfu polymerase. All plasmids were propagated in GT116 *E. coli *cells; a cell line specifically developed for the replication of hairpin containing plasmids (Invivogen). DNA was extracted (Hi-speed Maxi-prep Kit, Qiagen), quantitated in triplicate (Nanodrop) and sequence confirmed via automated sequencing.

### Pfu-based PCR screening and gel electrophoresis

As previously described [38], inserted donor fragments were screened by gel analysis of PCR amplicons made using primers that flanked the MCS; forward (5'-3'): AGT TCT GCA CTC GGC CTC TG, and reverse (5'-3'): CCA TGG TCT GCA GTC GCT AG. These were positioned 38 bp upstream and 21 bp downstream (inclusive). The optimized Pfu-based PCR screening method consisted of the primers (20 pmol each), 1 × Pfu Ultra II HS buffer (Stratagene), 3.5 mM MgCl_2 _(total), 10 mM dNTPs (each), ~ 10 ng of template, 2.5 μl DMSO (5%), 0.5 μl Pfu Ultra II HS (Stratagene), and H_2_O to a final volume of 50 μl. Each PCR was cycled at 1×: 95°C for 2 min., 35×: 95°C for 20 sec. | 66°C for 20 sec. | 72°C for 0.5 - 4 min. (depending upon template length), and 1× 72°C for 3 min. Samples were electrophoresed on 1% TAE agarose gels plus 0.01% SYBRSafe stain (Invitrogen) at 200 V (limiting) for ~ 60 min. using a 150 × 245 mm tray, 3 mm wells with a Bio-Rad sub-cell model 192 apparatus.

### Sequence confirmation

The sequence of each sub- and all final combinations were verified by automated sequencing, which was technically challenging due to high amounts of secondary structure resulting in poor reads, and the lack of unique binding sites due to repeated sequence shared amongst all cassettes. It required the use of the external primers previously described for the PCR screening method (binding to flanking plasmid regions) and shRNA specific primers (designed to overlap unique stem regions) to create contiguous partial sequence reads that could be assembled into single continuous reads using CodonCode Aligner for Mac OS × (CodonCode Corporation; http://www.codoncode.com).

### Fluorescent reporter assay

Each shRNA expression plasmid was co-transfected with two reporters; the corresponding target-specific GFP fusion and a non-specific AsRed-1 fusion. The non-specific reporter was typically an AsRed-1-Vpr fusion, since we selected no Vpr shRNA targets. Low passage no. HEK293a cells (sourced from the American Type Culture Collection) [ATCC:**CRL-1573**] were seeded at a density of 5 × 10^5 ^cells per well (6 well plates; 2 ml of medium). Cells were transfected 1 day later using 1 μg of total DNA (400 ng of shRNA expression plasmid, 300 ng of target plasmid and 300 ng of control plasmid) with 4 μl of Lipofectamine 2000 (Invitrogen) in OptiMEM (Invitrogen) to a total volume of 100 μl/well. For titration experiments up to 400 ng of shRNA expression plasmid was supplemented with the appropriate amount of the corresponding empty expression control plasmid to a total amount of 400 ng as indicated. Cells were analyzed by flow cytometry ~ 48 hours later (FACsCalibur and CellQuest Pro for Mac OS X, BD Biosciences). Target-specific suppression was measured as a decrease in green fluorescence (FL1 channel) and non-specific effects were measured as a change in red fluorescence (FL2 channel). The Fluorescence Index (FI) of cells in each channel was calculated by multiplying the geo mean of fluorescence by the percentage of cells that were fluorescent (only those cells gated above background). The FI of FL1 (green, target-specific activity) was normalized to remove non-specific effects (FI of FL1 normalized to the FI of FL2) and was expressed as a percentage of the FI of cells transfected only with the control plasmid that expressed no shRNA. Each experiment included a mock transfection (i.e. no DNA) and an off-target shRNA control (to verify that on-target shRNA suppression was sequence-specific), both of which behaved as expected and both of which were omitted from the graphs for clarity.

## Competing interests

This work was done by employees of Johnson and Johnson Research (JJR), for JJR.

## Authors' contributions

GJM and TLA conceived the experiments. GJM, JLG, YY and AT constructed the plasmids and performed the fluorescent reporter assays. GJM and TLA analyzed and interpreted the results. GJM. wrote the manuscript. All authors have read and approved the final manuscript.

## Supplementary Material

Additional file 1 **Contains additional data for the control sequences, detailed conservation profiles for the 10 selected shRNAs, detailed construction methods for the single shRNA expression plasmids and sequences for the reporter (mapped with the chosen shRNAs) and control sequences**. Click here for file
