# Supplementary material for: Multiple shRNA combinations for near-complete coverage of all HIV-1 strains
Source: AIDS Res Ther. 2011 Jan 13;8:1. doi: 10.1186/1742-6405-8-1 (PMC3033792; doi:10.1186/1742-6405-8-1)
Supplement: Additional file 1 — Contains additional data for the control sequences, detailed conservation profiles for the 10 selected shRNAs, detailed construction methods for the single shRNA expression plasmids and sequences for the reporter (mapped with the chosen shRNAs) and control sequences. [file 1742-6405-8-1-S1.PDF]

Supplementary Data

Combined activities for control sequences measured with an all-in-one reporter

We created and tested extra non-matched shRNA plasmids including single shRNA vectors plus **c**ontrol **c**ombinations (**c.c.**)). Each were separately transfected with the three all-in-one reporters; the HIV-specific aio sense and anti-sense reporters, and the non-matched control (though it *is* matched to the 7 control shRNAs shown here). Off-scale values (> 100 %, i.e. no activity) are indicated by open circles and text labels where appropriate. Values shown are representative of 2 or more independently repeated experiments.

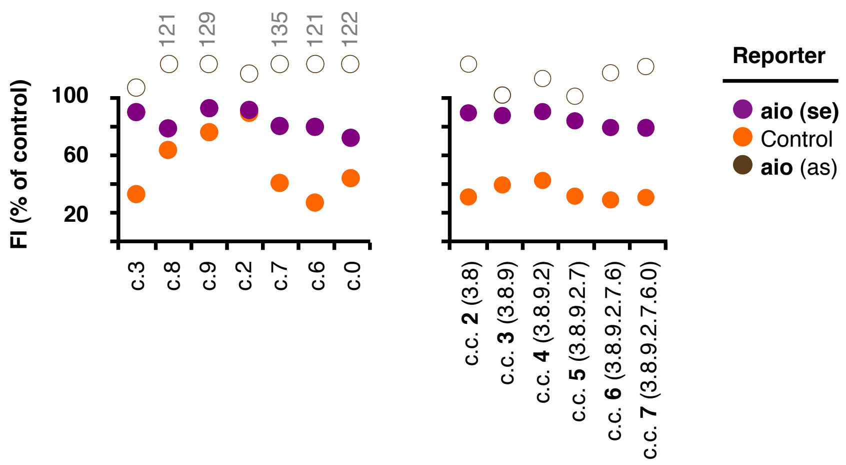

Supplementary materials and methods

Detailed conservation profiles for the 10 selected shRNAs

The following table shows the detailed conservation profiles for the 10 selected shRNAs calculated in our previous study. This information is an excerpt from Mcintyre et al. “96 shRNAs designed for maximal coverage of HIV-1 variants” **Retrovirology** (2009) vol. 6 (1) pp. 55. HIV-1 sequence data was compiled from 2 sources; publicly available sequence from the Los Alamos National Laboratory (LANL; www.hiv.lanl.gov) and proprietary sequence information from Virco (www.virco.com). The LANL data set included all near full-length genome sequences and gene sequence fragments as of December 2006. HIV-2 and SIV sequences were examined and excluded as they were sufficiently divergent to the NL4-3 HIV-1 reference strain [Genbank:**AF324493**]. The Virco data set was a small, but highly relevant private data set obtained from 105 HIV-1 infected persons from Europe. It contained only gene-specific sequences for the 6 accessory genes; Tat, Rev, Vif, Vpu, Vpr and Nef. Conservations were calculated for LANL and Virco sequences combined, LANL clade B subtypes only, and the Virco sequences only (where applicable). In each group, 6 conservations are given: the conservation for the entire overlapping 23 mer region, and the 5 individual conservations for the 5 overlapping 19 mers that make up the 23 mer profile region.

		LANL + Virco (all)						LANL clade B						Virco (all)					
**#**	**Target**	**23**	**-2**	**-1**	**p0**	**+1**	**+2**	**23**	**-2**	**-1**	**p0**	**+1**	**+2**	**23**	**-2**	**-1**	**p0**	**+1**	**+2**
0	LTR 510-21	69	69	69	**70**	71	71	100	100	100	**100**	100	100	-	-	-	-	-	-
1	LTR 527-21	90	93	93	**93**	93	91	100	100	100	**100**	100	100	-	-	-	-	-	-
2	Gag 533-20	58	62	70	**70**	69	71	77	78	80	**80**	80	82	-	-	-	-	-	-
3	Pol 248-20	75	80	80	**80**	79	80	77	81	87	**87**	86	87	-	-	-	-	-	-
4	Pol 2670-21	72	78	78	**73**	73	74	73	84	84	**74**	74	74	-	-	-	-	-	-
5	Pol 2878-20	74	84	76	**74**	74	74	79	91	80	**80**	79	79	-	-	-	-	-	-
6	Vif 9-21	71	71	71	**71**	71	71	91	91	91	**92**	92	92	74	75	75	**74**	74	74
7	Tat (x1) 140-21	68	81	82	**73**	72	72	73	79	78	**77**	75	75	62	77	80	**77**	74	73
8	Vpu 143-20	55	56	65	**66**	65	66	72	72	82	**83**	83	83	65	66	69	**69**	68	69
9	Env 1428-21	72	79	79	**73**	75	75	80	84	84	**81**	82	82	-	-	-	-	-	-
	**Average (all 10) :**	70	75	76	74	74	75	82	86	87	85	85	85	67	73	75	73	72	72

Constructing the single shRNA expression plasmids

The inserts for all of the shRNA expression plasmids (except shRNA #1) were built from a single synthetic oligonucleotide ~ 72 - 75 nt. long. Each oligonucleotide template consisted of a partial RE recognition site (*Bam* HI), the hairpin sense (or upper) sequence, a loop sequence, the hairpin anti-sense sequence, a pol III terminator sequence, and a second partial RE recognition site (*Hind* III), organized in the following general format: 5'-GCGCGGATCC | core(20 - 21) | NCTCGAGN | core(20 - 21) | [G/A/C] | TTTTTTGGAAGCTT-3'. A short primer sequence (12 nt.) common to the 3' end of all oligonucleotides was also designed (5'-CGCGAAGCTTCCAAAAAA-3'), annealed to each oligonucleotide template and extended with Phi-29 DNA polymerase in a single-step isothermal extension reaction. This step generated double-stranded synthetic inserts that were then digested to create 'sticky ends' and cloned as per standard procedures (as previously described). The template for shRNA #1 was created from standard complementary oligonucleotide pairs with offset ends as each shRNA core sequence contained an internal *Hind* III site making it incompatible with the Phi-29 extension method.

Phi-29 extension (shRNAs #0, #2 - #9)

Oligos were ordered at the minimal synthesis and purification scales (0.05 μM and desalt, Sigma-Genosys) and were re-suspended in water (1 - 10 μg / μl). Twenty picomoles of each oligo was used in the extension reaction (1x reaction buffer, 2x BSA, 20 μM dNTPs (1μl of a 10 mM stock), 10 units of Phi29 (New England Biolabs) and water to 20 μl), which was incubated at 30 °C for ~10 min., then 65 °C for 10 min. (to inactivate the polymerase). The extension product was digested (*Bam* HI plus *Hind* III), purified using the Nucleotide Removal kit (Qiagen), ligated to the expression plasmid and used to transform electrocompetent GT116 E.*coli*. Positive clones were confirmed by automated sequencing using our loop digestion method. Each single shRNA construct was digested and sequenced in two reactions, one containing the forward primer, the other containing the reverse. The primers bound to the expression plasmid backbone approximately 100 bases away from the region encoding the base of the hairpin stem. Each reaction contained: 1x RE buffer (NEB-2, New England Biolabs), 1x BSA, ~ 500 ng of template plasmid, 10 pmol of sequencing primer, ~ 10 units XhoI and water to a total volume of 16 μl, and was incubated at 37 °C for 30 – 60 min. prior to shipping, without purification, to an automated sequencing facility (Australian Genome Research Facility, AGRF).

Hairpin #	Target (NL4-3)	Oligonucleotide template (5' to 3')
0 (5)	LTR 510-21	GCGCGGATCCCCCACTGCTTAAGCCTCAATAACTCGAGATATTGAGG CTTAAGCAGTGGGTTTTTTGGAAGCTT
1 (8)	LTR 527-21	Overlapping oligonucleotide pair used instead, see below

Hairpin #	Target (NL4-3)	Oligonucleotide template (5' to 3')
2 (12)	Gag 533-20	GCGCGGATCCGAGCCACCCCACAAGATTTATCTCGAGTTAAATCTTGT GGGGTGGCTCTTTTTTGGAAGCTT
3 (22)	Pol 248-20	GCGCGGATCCGAGCAGATGATACAGTATTACCTCGAGCTAATACTGTAT CATCTGCTCTTTTTTGGAAGCTT
4 (36)	Pol 2670-21	GCGCGGATCCAGTACAAATGGCAGTATTCATACTCGAGAATGAATACT GCCATTTGTACTGTTTTTTGGAAGCTT
5 (43)	Pol 2878-20	GCGCGGATCCGGTGAAGGGGCAGTAGTAATTCTCGAGTATTACTACTG CCCCTTCACCTTTTTTGGAAGCTT
6 (51)	Vif 9-21	GCGCGGATCCCAGATGGCAGGTGATGATTGTACTCGAGAACAATCAT CACCTGCCATCTGTTTTTTGGAAGCTT
7 (60)	Tat (exon 1) 140-21	GCGCGGATCCATGGCAGGAAGAAGCGGAGACACTCGAGAGTCTCC GCTTCTTCCTGCCATATTTTTTGGAAGCTT
8 (68)	Vpu 143-20	GCGCGGATCCGAGCAGAAGACAGTGGCAATCCTCGAGCATTGCCAC TGTCTTCTGCTCTTTTTTGGAAGCTT
9 (83)	Env 1428-21	GCGCGGATCCTTGGAGAAGTGAATTATATAAACTCGAGATTATATAATTC ACTTCTCCAATTTTTTGGAAGCTT

n.b. The target positions are the co-ordinates within each gene of NL4-3.

Standard complementary oligonucleotide pairs (shRNA #1)

The template for shRNA #1 was made as a synthetic duplexes with overhanging ends identical to those created by restriction enzyme (RE) digestion (*Bam* HI at the 5' and *Hind* III at the 3'). The coding region for each hairpin was contained within a single oligonucleotide (upper oligo: 5'-GATCC | core(19-29) | NCTCGAGN | core(19-29) | [G/A/C] | TTTTTTGGA-3') and its complementary equivalent (lower oligo: 5'-AGCTTCCAAAAAA | [G/A/C] | core(19-29) | NCTCGAGN | core(19-29) | G-3'). Both oligos were synthesized and re-suspended as previously described. 1 μl from each was added to 98 μl of annealing solution (10 mM Tris-Cl pH 8.0, 50 mM NaCl, 1 mM EDTA), heated to 100 °C for 5 minutes, slowly equilibrated to room temperature and diluted up to 10,000 fold for ligation. The insert and plasmid were ligated, and used to transform electrocompetent GT116 E.*coli* (Invivogen).

Hairpin #	Target	Oligonucleotide template (5' to 3')
1 (8) (upper)	LTR 527-21	AGCTTTCCAAAAAACAATAAAGCTTGCCTTGAGTGCTCTCGAGTGCAC TCAAGGCAAGCTTTATTG
1 (8) (lower)	"	GATCCAATAAAGCTTGCCTTGAGTGCACTCGAGAGCACTCAAGGCAA GCTTTATTGTTTTTTGGA

The reporter and control sequences

The fluorescent reporters used in this study were assembled with following target domains derived from the common HIV-1 laboratory strain, NL4-3 [Genbank:**AF324493**]. n.b. each of the following target domains was fused immediately downstream of GFP with several stop codons placed between the domains.

Target-specific reporters

Nef-LTR (1255 bp): Nef shown in grey, LTR shown in UPPERcase

(matched to shRNA **#0** and **#1**)

tggaagggctaattcactcccaaagaagacaagatatccttgatctgtggatctaccacacacaaggctacttccctgattgg
cagaactacacaccagggccaggggtcagatatccactgacctttggatggtgctacaagctagtaccagttgagccagat
aaggtagaagaggccaataaaggagagaacaccagcttgttacaccctgtgagcctgcatggaatggatgaccctgagag
agaagtgttagagtggaggtttgacagccgcctagcatttcatcacgtggcccgagagctgcatccggagtacttcaagaac
tgctg**aA**TGGGTGGCAAGTGGTCAAAAAGTAGTGTGATTGGATGGCCTGCTGTAAGGGAAA
GAATGAGACGAGCTGAGCCAGCAGCAGATGGGGTGGGAGCAGTATCTCGAGACCTAGAA
AAACATGGAGCAATCACAAGTAGCAATACAGCAGCTAACAATGCTGCTTGTGCCTGGCTAG
AAGCACAAGAGGAGGAAGAGGTGGGTTTTCCAGTCACACCTCAGGTACCTTTAAGACCAA
TGACTTACAAGGCAGCTGTAGATCTTAGCCACTTTTTAAAAGAAAAGGGGGGACTGGAAG
GGCTAATTCACTCCCAAAGAAGACAAGATATCCTTGATCTGTGGATCTACCACACACAAGG
CTACTTCCCTGATTGGCAGAACTACACACCAGGGCCAGGGGTCAGATATCCACTGACCTT
TGGATGGTGCTACAAGCTAGTACCAGTTGAGCCAGATAAGGTAGAAGAGGCCAATAAAGG
AGAGAACACCAGCTTGTTACACCCTGTGAGCCTGCATGGAATGGATGACCCTGAGAGAGA
AGTGTTAGAGTGGAGGTTTGACAGCCGCCTAGCATTTCATCACGTGGCCCGAGAGCTGCA
TCCGGAGTACTTCAAGAACTGCTG**AC**ATCGAGCTTGCTACAAGGGACTTTCCGCTGGGGA
CTTTCCAGGGAGGCGTGGCCTGGGCGGGACTGGGGAGTGGCGAGCCCTCAGATGCTG
CATATAAGCAGCTGCTTTTTGCCTGTACTGGGTCTCTCTGGTTAGACCAGATCTGAGCCTG
GGAGCTCTCTGGCTAACTAGGGAA**CCCACTGCTTAAGCCTCaa**taaagcttgccttgagtGCTT
CAAGTAGTGTGTGCCCGTCTGTTGTGTGACTCTGGTAACTAGAGATCCCTCAGACCCTTTT
AGTCAGTGTGGAAAATCTCTAGCA

Gag-500 (500 - 1150) (650 bp)

(matched to shRNA **#2**)

aagtaatacccatgttttcagcattatcagaag**GAGCCACCCCACAAGATTT**aaataccatgctaaacacagtgg
ggggacatcaagcagccatgcaaatgttaaaagagaccatcaatgaggaagctgcagaatgggatagattgcatccagtg
catgcagggcctattgcaccaggccagatgagagaaccaaggggaagtgacatagcaggaactactagtacccttcagg
aacaaataggatggatgacacataatccacctatcccagtaggagaaatctataaaagatggataatcctgggattaaataaa

atagtaagaatgtatagccctaccagcattctggacataagacaaggaccaaaggaacccttagagactatgtagaccgat
tctataaaactctaagagccgagcaagcttcacaagaggtaaaaaattggatgacagaaaccttgttggtccaaaatgcgaa
cccagattgtaagactattttaaaagcattgggaccaggagcgacactagaagaaatgatgacagcatgtcagggagtggg
gggacccggccataaagcaagagttttggctgaagcaatgagccaagtaacaaatccagctaccataatgatacagaaag
gcaatttt

Pol-1 (1 - 436) (436 bp)

(matched to shRNA **#3**)

tttttagggaagatctggccttcccacaagggaaggccagggaattttcttcagagcagaccagagccaacagccccacc
agaagagagcttcaggtttggggaagagacaacaactccctctcagaagcaggagccgatagacaaggaactgtatccttt
agcttccctcagatcactctttggcagcgacccctcgtcacaataaagatagggggggcaattaaaggaagctctattagatac
ag**GAGCAGATGATACAGTATT**agaagaaatgaatttgccaggaagatggaaaccaaaaatgatagggggaatt
ggaggttttatcaaagtaagacagtatgatcagatactcatagaaatctgcggacataaagctataggtacagtattagtagga
cctacacctgtcaacataattggaagaaatctgt

Pol-2670 (41 bp)

(matched to shRNA **#4**)

ttaagacagc**AGTACAAATGGCAGTATTC**atccacaatttt

Pol-2878 (41 bp)

(matched to shRNA **#5**)

cctctggaaa**GGTGAAGGGGCAGTAGTAA**tacaagataata

Vif (579 bp)

(matched to shRNA **#6**)

Atggaaaa**CAGATGGCAGGTGATGATT**gtgtggcaagtagacaggatgaggattaacacatggaaaagattag
taaaacaccatatgtatatttcaaggaaagctaaggactggttttatagacatcactatgaaagtactaatccaaaaataagttc
agaagtacacatcccactaggggatgctaaattagtaataacaacatattggggtctgcatacaggagaaagagactggcat
ttgggtcagggagtctccatagaatggaggaaaaagagatatagcacacaagtagaccctgacctagcagaccaactaatt
catctgcactattttgattgtttttcagaatctgctataagaaataccatattaggacgtatagttagtcctaggtgtgaatatcaagc
aggacataacaaggtaggatctctacagtacttggcactagcagcattaataaaaccaaaacagataaagccacctttgcct
agtgttaggaaactgacagaggacagatggaacaagccccagaagaccaagggccacagagggagccatacaatgaat
ggacactag

Tat x12 (261 bp)

(matched to shRNA **#7**)

atggagccagtagatcctagactagagccctggaagcatccaggaagtcagcctaaaactgcttgtaccaattgctattgtaa
aaagtgttgctttcattgccaagtttgtttcatgacaaaagccttaggcatctcct**ATGGCAGGAAGAAGCGGAG**ac
agcgacgaagagctcatcagaacagtcagactcatcaagcttctctatcaaagc**aa**cccacctcccaatcccgaggggac
ccgacaggcccgaaggaatag

Vpu (246 bp)

(matched to shRNA **#8**)

atgcaacctataatagtagcaatagtagcattagtagtagcaataataatagcaatagttgtgtggtccatagtaatcatagaata taggaaaatattaagacaaagaaaaatagacaggttaattgatagactaatagaaa**GAGCAGAAGACAGTGGC AA**tgagagtgaaggagaagtatcagcacttgtggagatgggggtggaaatggggcaccatgctccttgggatattgatgat ctg**tag**

Env-1300 (1300 - 1740) (440 bp)

(matched to shRNA **#9**)

cccctcccatcagtggacaaattagatgttcatcaaatattactgggctgctattaacaagagatggtggtaataacaacaatg ggtccgagatcttcagacctggaggaggcgatatgagggacaa**TTGGAGAAGTGAATTATAT**aaatataaagta gtaaaaattgaaccattaggagtagcacccaccaaggcaaagagaagagtggtgcagagagaaaaaagagcagtggga ataggagctttgttccttgggttcttgggagcagcaggaagcactatgggctgcacgtcaatgacgctgacggtacaggcca gacaattattgtctgatatagtgcagcagcagaacaatttgctgagggctattgaggcgcaacagcatctgttgcaactcaca gtctggggcatcaaacagctccaggcaagaatcctggct

All-in-one reporters

The **aio** reporters were created using custom generated target domains (GenScript; www.genscript.com) comprised of 9 target domains matched to our 10 shRNAs, or 7 un-matched control domains (for the control reporter) which were transferred via PCR into our EGFP reporter base plasmids. The sequence of the 406 bp combined target domain syn-thesized for the aio sense reporter was (*Xho* I - *Bgl* II {*for cloning*} - #9 - #2 - #0|1 - #3 - #4 - #5 - #7 - #6 - #8 - *Sal* I - *Bam* HI {*for cloning*}) (5'-3'): ctcgag {*Xho* I} agatct {*Bgl* II} tgaggga-caaTTGGAGAAGTGAATTATAT**aa**atataaagta {#9, 21} ttatcagaagGAGCCACCCCACAA-GATTT**a**aataccatgc {#2, 20} aactagggaaCCCACTGCTTAAGCCTCAA**TA**AAGCTTGCCTTGAGT**gc**ttcaagtagt {#0, 21 | #1, 21} ttagatacagGAGCAGATGATACAGTATT**a**gaagaaatga {#3, 20} ttaagacagcAGTA-CAAATGGCAGTATTC**at**ccacaatttt {#4, 21} cctctggaaaGGTGAAGGGGCAGTAGTAA**t**acaa-gataat {#5, 20} ggcatctcctATGGCAGGAAGAAGCGGAG**ac**agcgacgaag {#7, 21} ccatggaaaaCAGATGGCAGGTGATGATT**gt**gtggcaagta {#6, 21} ctaatagaaaGAGCAGAAGA-CAGTGGCAA**t**gagagtgaag {#8} gtcgac {*Sal* I} ggatcc {*Bam* HI} (gaps shown between fea-tures for clarity, the p0 19 mer shRNA core sites in UPPERcase, and the extra 1 - 2 (3') sites of the shRNA 20 - 21 bp stem shown in **bold**). The unbroken sequence for the 3 aio report-ers was:

aio-sense (406 bp)

ctcgagagatcttgagggacaattggagaagtgaattatataaatataaagtattatcagaaggagccaccccacaagattta aataccatgcaactagggaacccactgcttaagcctcaataaagcttgccttgagtgcttcaagtagtttagatacaggagca gatgatacagtattagaagaaatgattaagacagcagtacaaatggcagtattcatccacaattttcctctggaaaggtgaagg

ggcagtagtaatacaagataatggcatctcctatggcaggaagaagcggagacagcgacgaagccatggaaaacagatg
gcaggtgatgattgtgtggcaagtactaatagaaagagcagaagacagtggcaatgagagtgaaggtcgac**ggatcc**

aio-antisense (406 bp)

ctcgaggtcgaccttcactctcattgccactgtcttctgctctttctattagtacttgccacacaatcatcacctgccatctgttttc
catggcttcgtcgctgtctccgcttcttcctgccataggagatgccattatcttgtattactactgcccсttcacctttccagagga
aaattgtggatgaatactgccatttgtactgctgtcttaatcatttcttctaatactgtatcatctgctcctgtatctaaactacttgaag
cactcaaggcaagctttattgaggcttaagcagtgggttccctagttgcatggtatttaaatcttgtggggtggctccttctgataa
tactttatatttatataattcacttctccaattgtccctcaagatct**ggatcc**

non-matched control (308 bp)

ctcgagagatctatgaaatataaatatattaagtgaagaggttaacagggagtcgtaccataaatttagaacaccccaccga
ggaagactatttccgttcgaaataactccgaattcgtcacccaagggatcaaagtaaagaagattatgacatagtagacgag
gacatagattgaagcagcgacagaggcgaagaaggacggtatcctctacggatgaacggtgtgttagtagtggacggtag
acaaaaggtaccgaagtgagagtaacggtgacagaagacgagaaagataatcgtcgac**ggatcc**

Oligonucleotide templates for the off-target and non-matched control shRNAs

The off-target hairpin plasmid (omitted from all graphs for clarity) was taken from previous work, but was originally constructed using standard complementary oligonucleotide pairs as described above. The following table lists the corresponding complementary oligonucleotide pairs, with the stem region indicated in light grey.

Hairpin #	Target	Oligonucleotide template (5' to 3')
off target (upper)	none	GATCCAAGACAGTCCAACACACGCCACCTGTCTCTCTCGAGTGAGA CAGGTGGCGTGTGTTGGACTGTCTTTTTTTTGGA
off target (lower)	"	AGCTTTCCAAAAAAAAGACAGTCCAACACACGCCACCTGTCTCACTC GAGAGAGACAGGTGGCGTGTGTTGGACTGTCTTG

The 7 non-matched shRNA controls used to make the **2** (3.8), **3** (3.8.9), **4** (3.8.9.2), **5** (3.8.9.2.7), **6** (3.8.9.2.7.6) and **7** (3.8.9.2.7.6.0) cassette control plasmids were derived from the backwards complement of shRNAs #3, #8, #9, #2, #7, #6 and #0. In this way they were unmatched to the aio reporters yet had identical nucleotide compositions (but in reverse order) to retain similar thermodynamic profiles. The following table lists the sense (upper) strand core sequences for the 7 non-matched controls.

**The original HIV-1 shRNAs**		**The corresponding non-matched controls**	
**shRNA #**	**Matched shRNA p0 core (5' to 3')**	**control #**	**Backwards controls (5' to 3')**
0	CCCACTGCTTAAGCCTCAA	c0	ATAACTCCGAATTCGTCACCC
2	GAGCCACCCCACAAGATTT	c2	ATTTAGAACACCCCACCGAG
3	GAGCAGATGATACAGTATT	c3	ATTATGACATAGTAGACGAG
6	CAGATGGCAGGTGATGATT	c6	TGTTAGTAGTGGACGGTAGAC
7	ATGGCAGGAAGAAGCGGAG	c7	CAGAGGCGAAGAAGGACGGTA
8	GAGCAGAAGACAGTGGCAA	c8	TAACGGTGACAGAAGACGAG
9	TTGGAGAAGTGAATTATAT	c9	AATATATTAAGTGAAGAGGTT
